# Local Sourcing and Supplier Development in Global Health: Analysis of the Supply Chain Management System's Local Procurement in 4 Countries

**DOI:** 10.9745/GHSP-D-18-00083

**Published:** 2018-10-03

**Authors:** Prashant Yadav, Sarah Alphs, Clinton D'Souza, Gordon Comstock, Iain Barton

**Affiliations:** a Department of Global Health and Social Medicine, Harvard Medical School, Boston, MA, USA.; b Independent researcher, Dar es Salaam, Tanzania.; c Imperial Health Sciences and Partnership for Supply Chain Management, Bedfordview, Gauteng, South Africa.; d Management Sciences for Health and Partnership for Supply Chain Management, Arlington, VA, USA.

## Abstract

From 2006 to 2014, Supply Chain Management System (SCMS), the global procurement and distribution project for the U.S. President's Emergency Plan for AIDS Relief (PEPFAR), distributed over US$1.6 billion worth of antiretroviral drugs and other health commodities, with over US$263 million purchased from local vendors in 14 countries in sub-Saharan Africa. A simple framework was developed and 39 local suppliers from 4 countries were interviewed between 2013 and 2014 to understand how SCMS local sourcing impacted supplier development. SCMS local suppliers reported new contracts with other businesses (77%), new assets acquired (67%), increased access to capital from local lending institutions (75%), offering more products and services (92%), and ability to negotiate better prices from their principles (80%). Additionally, 70% (n=27) of the businesses hired between 1 and 30 new employees after receiving their first SCMS contract and 15% (n=6) hired between 30 and 100 new employees. This study offers preliminary guidance on how bilateral and multilateral agencies could design effective local sourcing programs to create sustainable local markets for selected pharmaceutical products, laboratory, and transport services.

## Abstract (precis)

Local suppliers reported that after doing business with PEPFAR's global procurement and distribution project for essential HIV/AIDS medicines and supplies, they achieved revenue and asset growth, improved their quality standards, acquired new contracts with other businesses, and hired more employees.

## BACKGROUND

Amidst a growing concern about sustainability of global health aid, and in a context of increasing focus on aid-recipient countries taking over their health budgets, the development of national program capacity has been attracting considerable policy interest.^1^^–^^5^ There is a strong interest in identifying viable institutional frameworks and policy options that can help countries graduating from foreign development assistance for health to effectively manage their health systems with local implementing partners.^2^ Many donors and development agencies, especially the United States Agency for International Development (USAID), have an increased focus on how to make programs more effective, more enduring, and less costly through procurement reform, sustainable partnerships, and local solutions.

This imperative for greater sustainability and higher value for money is most pronounced in the procurement and distribution programs run as part of global health initiatives such as the U.S. President's Emergency Plan for AIDS Relief (PEPFAR); the Global Fund to Fight AIDS, Tuberculosis, and Malaria; and Gavi, the Vaccine Alliance. USAID had initiated a strategy to increase local procurement with an intent to promote sustainability and country ownership of its programs.^6^ These trends are based on a hypothesis that local procurement—the practice of purchasing goods and/or services through local suppliers—can help achieve sustainability and build the capacity of local market actors and institutions.

In 2005, USAID under PEPFAR established the Supply Chain Management System (SCMS) to provide a reliable, cost-effective, and secure supply of products for HIV/AIDS programs in PEPFAR-supported countries. Between 2006 and 2014, SCMS distributed US$1.6 billion worth of antiretroviral drugs and other health commodities to various countries. One of the cornerstones of SCMS's mission is a commitment to developing the procurement and distribution capacity of host-country governments and local NGOs, and selecting the most appropriate procurement strategies for each product and geography. In tune with this strategy, SCMS sourced over US$263 million of its overall spending during this period from local suppliers in 14 African countries.

When commercial companies seek local suppliers, the reason is typically to reduce costs and meet local content requirements.^7^^,^^8^ While local purchasing by global multinationals has shown the benefits of lowering costs and achieving economic benefits,^6^ local procurement may not work for all product and geographical markets. In low- and middle-income country settings, the number of local suppliers who can meet the standard for quality and reliability required for producing pharmaceuticals and laboratory supplies are extremely limited. As a result, intermediaries who add cost markup without contributing to value addition sometimes get added into the value chain. While increased cost was of some concern, the key goals of the SCMS local sourcing initiative were not necessarily cost savings but sustainability and local capacity enhancement.

Global commercial companies often seek local suppliers to reduce costs and meet local production standards.

The goal of this study was to understand the impact of SCMS local sourcing on long-term development of a high-quality local supplier base. While supplier development has been extensively studied in the commercial sector^9^ and to some extent in high-income country public-sector procurement,^10^ it has not been studied systematically for public/NGO purchasers operating in international health settings. This study aimed to understand how local sourcing impacts supplier development and long-term market health. Our contribution sheds new light on how bilateral and multilateral agencies could design effective local sourcing programs to create sustainable local markets for selected pharmaceutical products, laboratory, and transport services.

## METHODOLOGY

The commonly used methodology in economic literature to assess broader intersectoral economic impact of an intervention is Input-Output Analysis.^11^ Input-Output Analysis looks at the interdependencies of industries or economic sectors and estimates to what extent positive or negative economic shocks affect a country's economy.^11^ While this type of analysis provides a useful way to assess overall economic impact, it does not capture firm specific managerial impacts that are at the core of our study. Another important way to assess impact is the concept of Shared Value,^12^^,^^13^ which assesses the gains to each stakeholder. Our study objectives were to understand the strategic, managerial, and economic implications for organizations that were part of the SCMS local vendor network. We developed a simple framework of measurement that was clear, practical, and grounded in the country context where this work was carried out.

**FIGURE 1:**
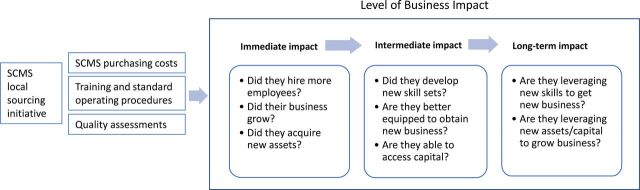
Impact Assessment Framework

The most direct impact measured was the increase in business revenue for the suppliers when SCMS procures a product or service from them. This revenue increase leads to further economic activity, including the creation of additional jobs at the supplier. Furthermore, as suppliers expand to meet additional or new needs, they potentially acquire additional assets, such as a warehouse, a forklift, or laboratory equipment. In addition, as part of the local sourcing initiative, SCMS provided training, helped create standard operating procedures, and enforced strict quality standards on the suppliers. This could improve suppliers' ability to better serve other customers and increase their competitiveness in seeking business with other companies. A clear predictable revenue stream from SCMS may also enable suppliers to access capital at better terms from banks and financial institutions. We used a 3-part framework of potential direct, intermediate, and long-term impact to assess the overall impact of SCMS on its suppliers (Figure 1) and surveyed sampled firms to help answer the 8 questions embedded within the framework. We measured this using the indicators described in the Table.

**TABLE.** Indicators for Impact Assessment

Impact	Indicator	Data Source
Revenue	Annual revenue pre-/post-SCMS award	Supplier company records
Business output/productivity	# training programs conducted Good storage practices Access to working capital	Supplier company records and interview
Assets (e.g., vehicles)	# of assets obtained/sold locally	Supplier company records
Employment	# of additional jobs created post-SCMS award	Supplier company records
Market competitiveness	# of contracts obtained post-SCMS award	Supplier company records and interview
Range of services offered	# and types of services offered post-SCMS award	Supplier interview

Abbreviation: SCMS, Supply Chain Management System.

Through their relationship with SCMS, local suppliers benefitted from increased revenue, additional resources to expand operations, and received training and technical support to help their businesses improve and grow.

We developed a survey and interview guide to assess these and additional changes in the supplier's economic position, relationships, and capacity over time (Supplement). The methodology did not include any control group—specifically, local firms that did not receive a SCMS contract—and was strictly based on comparisons before and after receiving a contractual award from SCMS.

### Sample Selection

Analysis of SCMS spending revealed that more than 90% of SCMS spending with local vendors between fiscal years 2009 and 2011 was in 8 countries (of 14): Côte d'Ivoire, Ethiopia, Kenya, Mozambique, Nigeria, South Africa, Tanzania, and Zambia. We sampled 50% of the 8 countries based on logistical/geographical clustering. The study was carried out in Ethiopia, Kenya, Mozambique, and Tanzania. Instead of interviewing and obtaining records from every single vendor in each of these countries, the below was used as inclusion criteria for vendors:

1. Contract award value over US$15,000.
2. Supplied at least 1 order to SCMS between 2006 and 2013.
3. Representation from different industries, such as laboratories, pharmaceutical, information and technology services, logistics, and warehousing.
4. Representation of both smaller and larger businesses.

A total of 39 vendors across all 4 countries were interviewed. The interviews were carried out over the period of May 2013 through January 2014.

### Profile of Local Suppliers

The local suppliers spanned from small vendors of office supplies or maintenance services to large pharmaceutical and laboratory supply companies. Before delving into understanding the impact of local procurement, it is necessary to understand who the local suppliers are, how long they have been in business, and how many employees they have.

Local suppliers ranged from small office suppliers to large pharmaceutical or laboratory companies.

#### Job Function of Respondents

Survey- and interview-based business impact studies often rely on interviews with line staff who have a limited understanding of strategic issues, such as the development of new service offerings. For this study, the research team interviewed respondents with managerial and strategic experience who were at the leadership level at each supplier. More than 20 of the respondents were chief operating officers or owners of the firm, which gave us an understanding of how local sourcing has transformed their business.

#### Industry of Local Supplier

**FIGURE 2:**
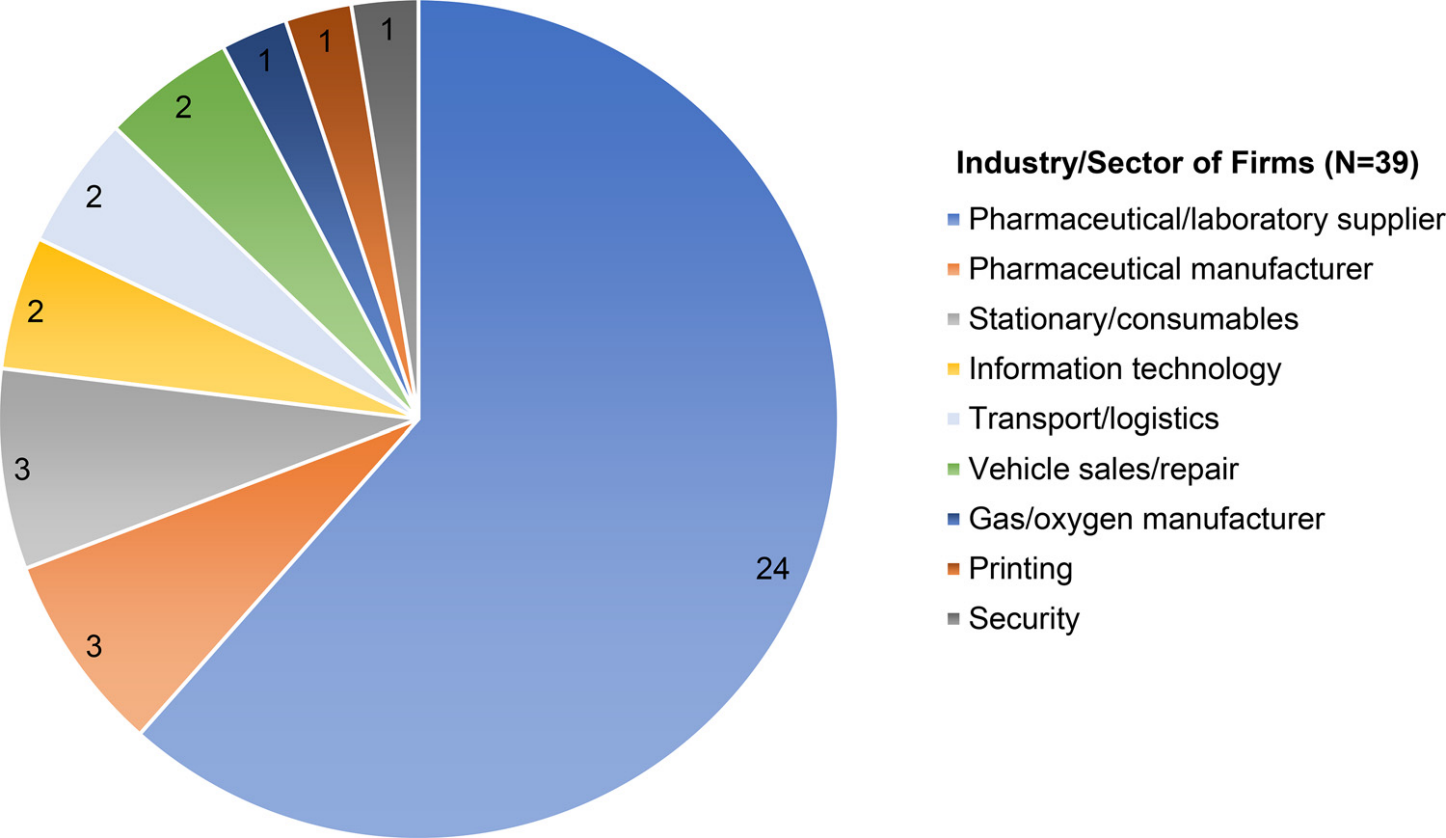
Industry/Sector of Firms in the Sample

A large portion of money spent at the local level was on suppliers of pharmaceuticals and laboratory services or reagents. Of the vendors interviewed (N=39), 24 were suppliers of laboratory, medical supplies, and pharmaceuticals (Figure 2).

#### Age of Business

Of the 39 local suppliers interviewed, 27 had been in operation for 5 years or more at the time of the interview. This demonstrates that a majority of the local vendors are established firms and are not new players that have created only to serve SCMS.

#### Size of Business

**FIGURE 3:**
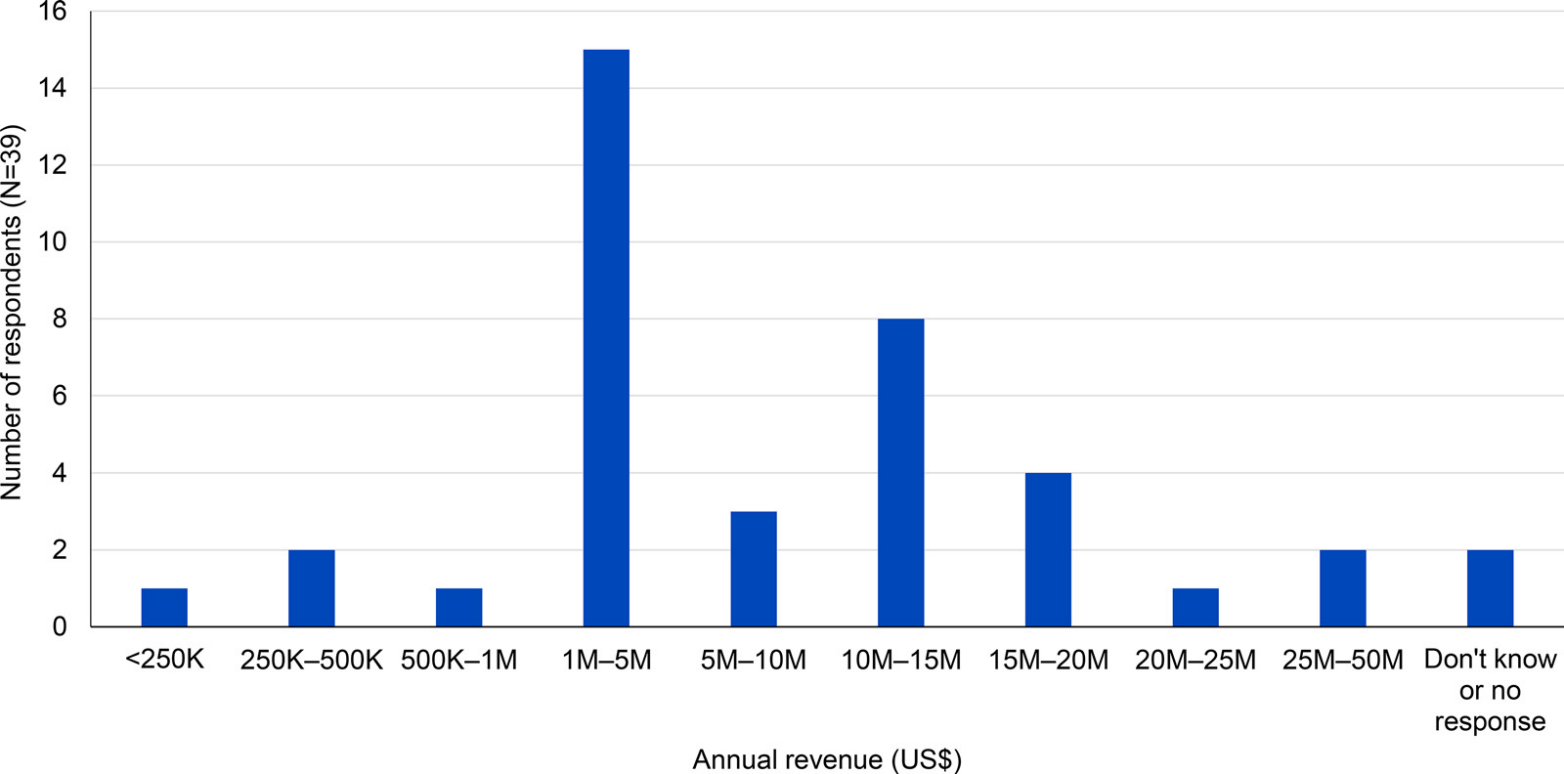
Annual Revenue (in US$) of Firms in the Sample

The reported median annual revenue of local suppliers was between US$1 million and US$5 million, with some earning as high as US$30 million through US$49.9 million (Figure 3).

The distribution of the size of the businesses varied considerably by the industry. All of the pharmaceutical and laboratory suppliers (n=24) reported an annual revenue greater than US$1 million, with many earning up to and over US$20 million. Stationary suppliers tended to be smaller, with 2 of them having revenue less than US$500,000 each year. Vendors supplying vehicles, including forklifts and trucks, were the largest vendors by type, with 2 reporting an annual revenue of over US$10 million. As noted in the sampling framework, only local businesses with a contract award over US$15,000 were included in the study, which slightly biased this sample toward larger companies.

#### Number of Employees

**FIGURE 4:**
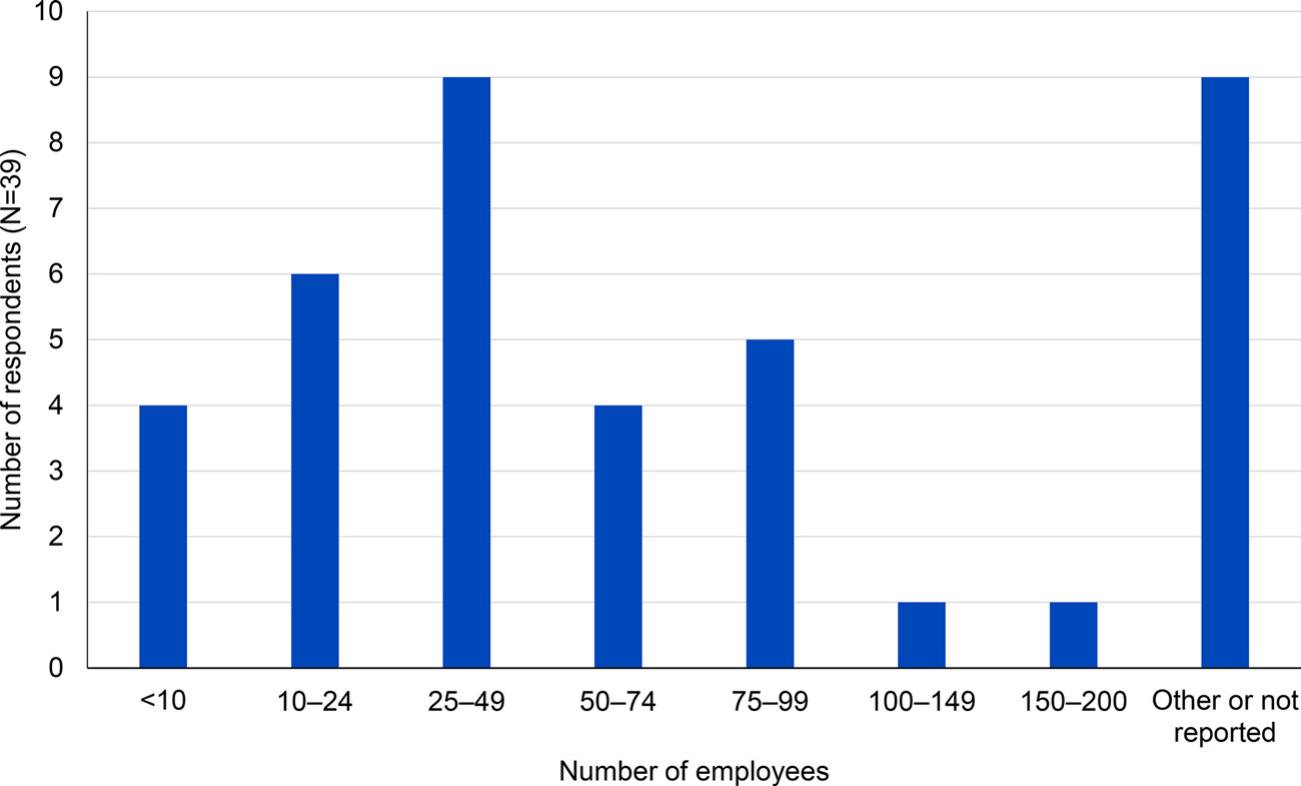
Number of Employees in Respondent Firms

SCMS local vendors interviewed included a balanced mix of smaller businesses with less than 25 employees and larger businesses with over 200 employees (Figure 4).

#### Branches

Of the 39 businesses interviewed, 24 said they had a branch in another location. Multi-locational businesses have a larger footprint, allowing them to provide services not only in the capital city but also in other parts of the country.

#### Number of Customers

The sampled businesses represented a balanced mix of companies that ranged from having less than 50 customers to having more than 500.

#### Vendor's Total SCMS Contract Award Values

**FIGURE 5:**
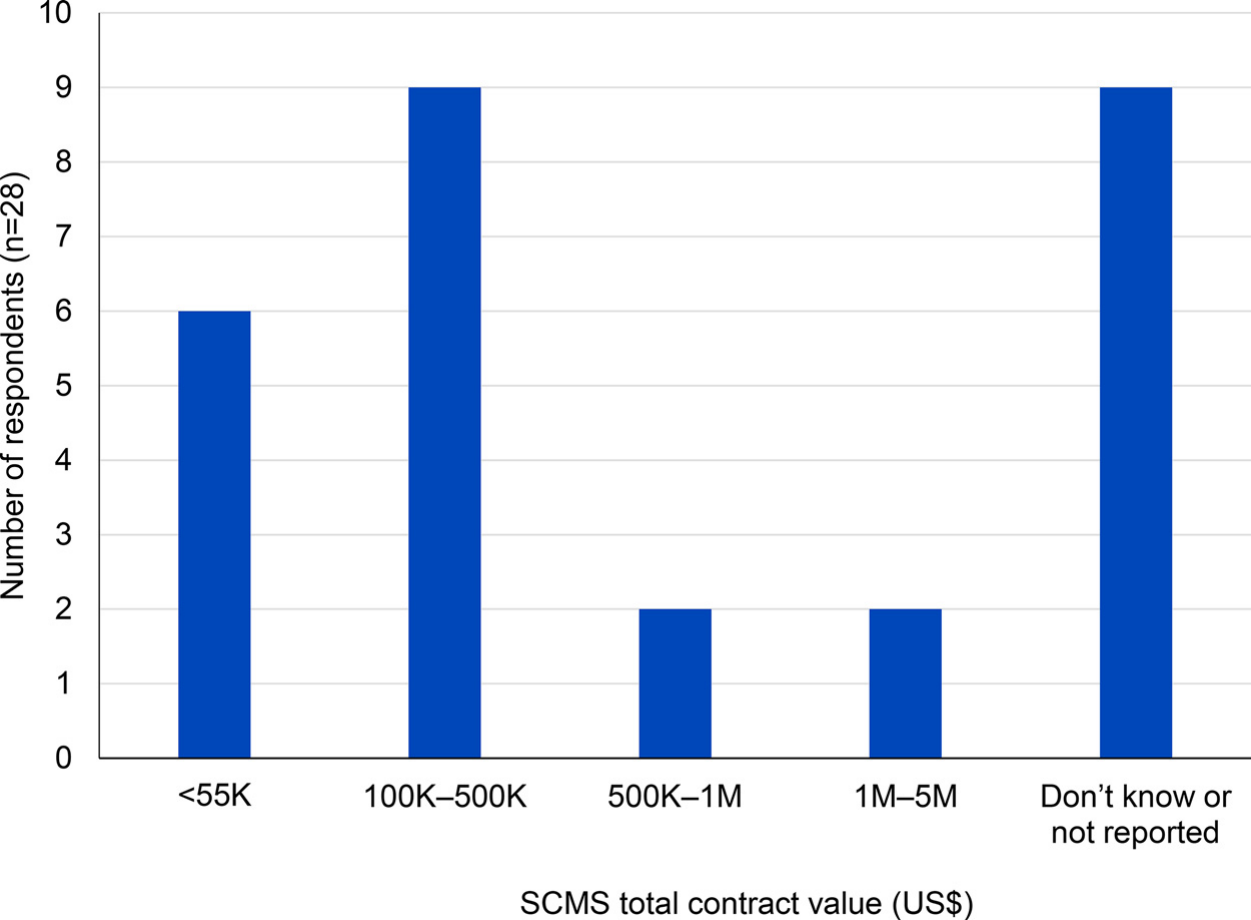
Distribution of SCMS Total Contract Value (US$) to Firms in the Sample Abbreviation: SCMS, Supply Chain Management System. Note: Kenya respondents were not included. When provided a range, the average of the range is included in this analysis.

SCMS provided the research team with the values of contracts awarded to vendors in Ethiopia, Mozambique, and Tanzania. Among these vendors (n=28), 26 were awarded contracts with a total value over US$100,000. The majority of vendors received contract awards valued between US$100,000 and US$500,000 (Figure 5).

The distribution of contract value varies considerably by the industry in which local vendors operate. In total, 3 manufacturers received contracts valued over US$500,000; 8 of the pharmaceutical and laboratory suppliers (distributors) received contracts between US$100,000 and US$500,000, 7 received contracts valued between US$500,000 and over US$10 million, and 1 received a contract valued less than US$50,000; and 2 of the suppliers supplying stationary and other office consumables received contracts valued less than US$50,000.

## RESULTS

### Revenue Growth

Almost all (n=36) of the 39 businesses interviewed reported that their company had experienced growth since their first contract with SCMS.

### Labor Changes and Employment Generation

**FIGURE 6:**
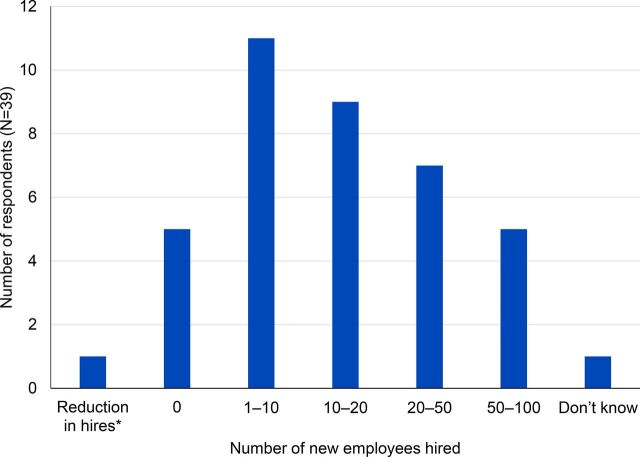
Number of New Employees Hired After Contract With SCMS Abbreviation: SCMS, Supply Chain Management System. * 1 firm reported a decrease in their employees after the start of SCMS contract.

A majority (n=33) of the firms reported an increase in the number of employees after receiving a contract from SCMS. Of the 33 firms, 27 hired between 1 and 30 new employees after receiving their first SCMS contract and 6 hired between 30 and 100 new employees (Figure 6). Employee salaries have a trickle down/multiplier effect on the local economy.

A majority (84.6%) of the firms reported an increase in the number of employees after receiving a contract from SCMS.

### Asset Growth

**FIGURE 7:**
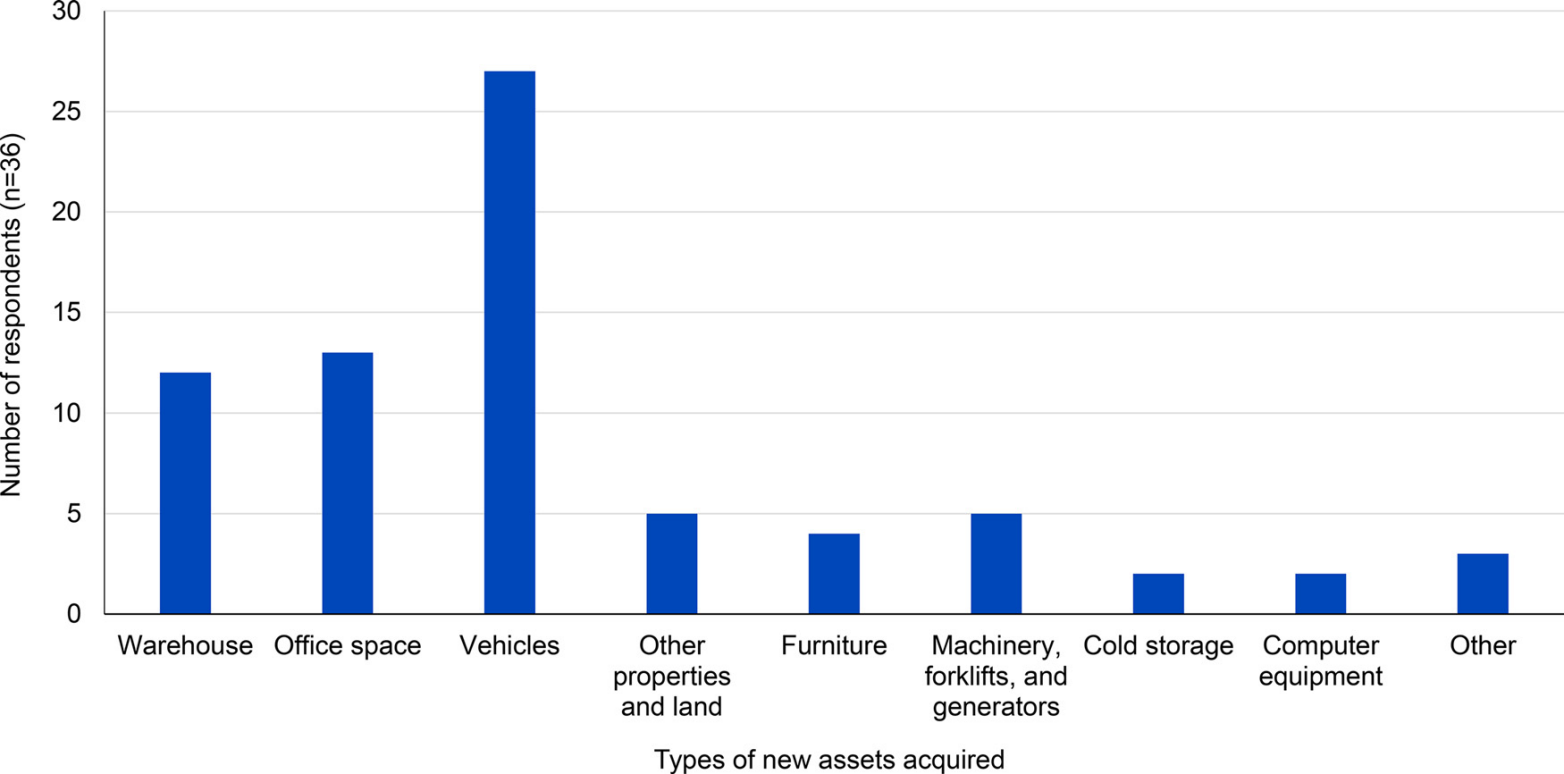
Types of New Assets Acquired^a^ After Start of SCMS Contract Abbreviation: SCMS, Supply Chain Management System. ^a^ More than 1 response was allowed.

**FIGURE 8:**
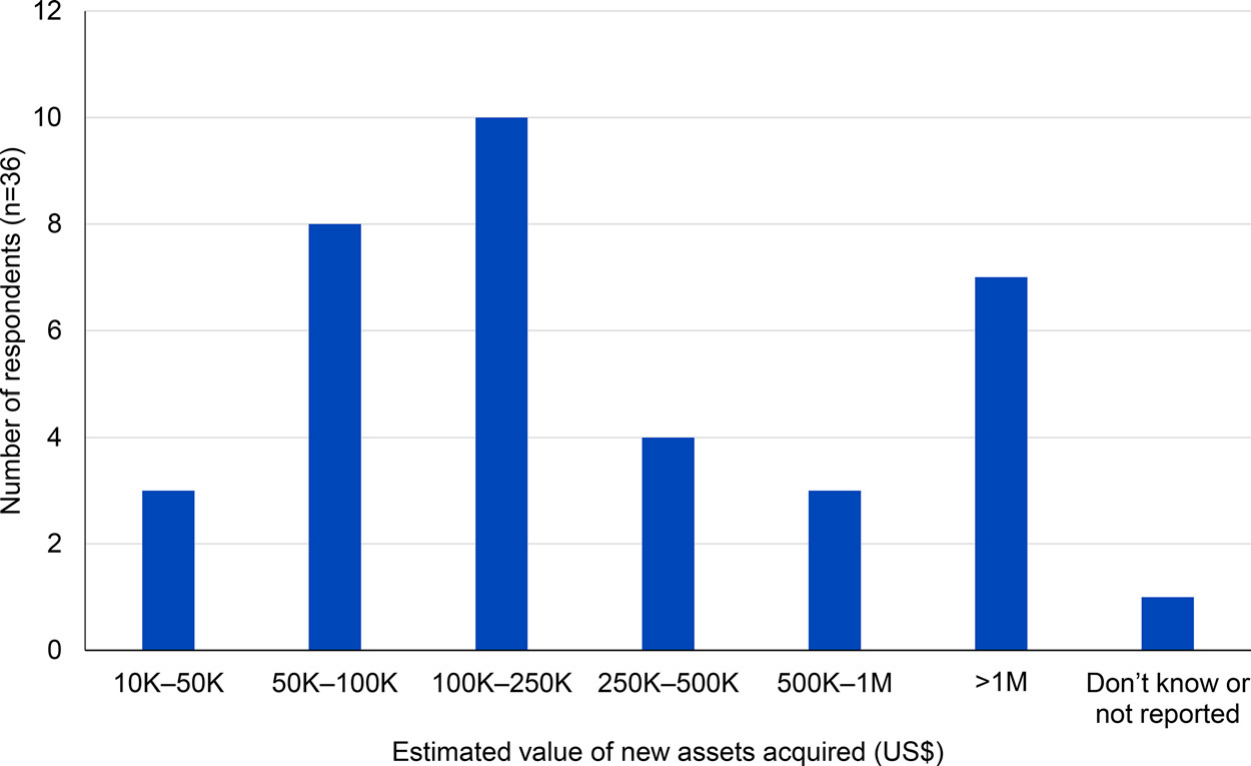
Estimated Value (in US$) of New Assets Acquired

A majority (n=36) of the 39 firms interviewed reported an increase in asset ownership after their first SCMS contract. The most commonly acquired assets were warehouse space, commercial vehicles, and additional office space (Figure 7). These are generic assets that can be effectively leveraged for other clients. The majority (n=25) of respondents reported acquiring new assets over US$100,000 (Figure 8).

### Overall Impact of SCMS Business

**FIGURE 9:**
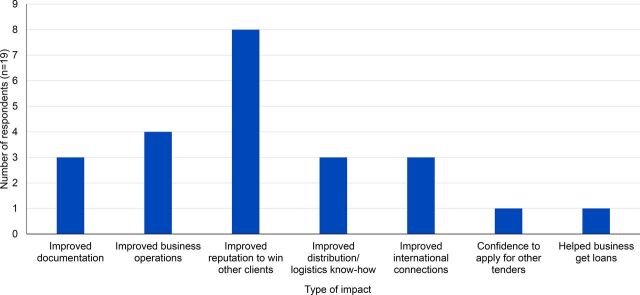
Type of Impact^a^ of SCMS Contract on Firm's Business Abbreviation: SCMS, Supply Chain Management System. ^a^ More than 1 response was allowed.

When the respondents were asked to reflect on the impact of SCMS on their businesses, about half (n=19) of 39 businesses interviewed said that SCMS had positively impacted their business, 2 reported that working with SCMS negatively impacted their business, 3 reported both positive and negative impacts, and 9 said that SCMS had no impact on their business (Figure 9). Of the 19 firms who reported that a contract with SCMS had a positive impact on their business, 8 stated that doing business with SCMS improved their reputation, enabling them to win new clients.

About half (48.7%) of 39 businesses interviewed said that SCMS had positively impacted their business.

Of the 2 vendors who reported that working with SCMS negatively impacted their business, the first cited that delayed payments from SCMS constrained their company financially and the second reported that working with SCMS had negatively impacted their company's reputation when facility-level stock-outs of a product they had supplied to SCMS were attributed to their business.

A total of 9 vendors responded that SCMS had no impact on their business. We analyzed these vendors in greater detail to understand the underlying reasons for their lack of growth. We found that nearly all (n=7) of the firms did not have any active contracts with SCMS in 2013 and that their earlier contracts were mostly one-off procurement arrangements. Of the 2 remaining firms, 1 was in financial distress with significant debt due to outstanding payments from large clients, including the national government, and did not attribute its financial issues to SCMS. The other was a large well-established business that had received its first contract with SCMS in 2013, but had many larger clients than SCMS.

## DISCUSSION

When large global firms source from local suppliers it provides a powerful channel through which the global firm's knowledge and skills can be diffused directly to the local suppliers, or indirectly to local firms through a spillover effect.^7^^,^^14^^,^^15^ However, sometimes it is difficult or impossible to find local suppliers who meet the international quality standards or capability requirements needed to expand the supply chain. Supplier development should be a key part of public and NGO procurement in such markets.

The results of this study show that, in most cases, local suppliers under the SCMS local sourcing initiative increased employment, achieved revenue and asset growth, improved their quality standards, and became more equipped to acquire new customers. Admittedly, some of this growth would have occurred due to overall growth in the economy/sector. Furthermore, firms with effective business processes, strong management, good governance, and a past record of growth were more likely to grow faster than their peers. Given SCMS's selection criterion for local vendors favors firms with such attributes, it became harder to establish to what extent their growth was due to SCMS contracts and know-how and how much would have occurred irrespective of those 2 factors.

Local suppliers need capital to continually upgrade the quality, breadth, and depth of the services they offer. In order to meet global standards and further grow their businesses, local suppliers require sufficient capital to invest in buildings, machinery, equipment, trucks, and product inventory. High interest rates, lack of longer-term loans, and the absence of credit records hinder the ability of small- and medium-sized suppliers to survive and grow. About a quarter (n=29) of the 39 local SCMS vendors in this study reported that their access to capital from local lending institutions increased after they began doing business with SCMS. This shift results from the predictability of demand and greater confidence by local banks in suppliers with track record of doing business with a large international firm. Evidence shows that alleviating working capital constraints substantially enhances business sustainability.^16^ To that end, supplier financing solutions that can provide greater access to capital and further enhance the growth of local suppliers need to be explored. Commercial companies often conduct a supplier sustainability gap analysis, which helps to identify the constraints current suppliers are facing in leveraging their enhanced capabilities to further grow and diversify their business. This type of analysis can help bilateral and multilateral agencies, host country governments, and their implementing partners identify opportunities and work together to design local supplier development programs. A key challenge to this approach is that when local suppliers are uncompetitive, forcing local sourcing through “local content requirements” can lead to high-cost production of low-quality products. A more appropriate policy intervention is to create investment vehicles that allow stronger linkages through which productivity know-how can be diffused from global firms to local companies.

A supplier sustainability gap analysis helps commercial companies identify the constraints faced by their suppliers to further grow their business.

Our findings show that local supplier development of smaller firms would require the buying group to provide a support role through coaching, mentoring, and other methods of transferring business know-how. For more established local firms (such as the ones in this study sample), the buying group's key role is their ability to provide predictable demand, guaranteed financial flows, and the reputational benefits of doing business with a large global firm (Figure 9). Local supplier development is an effort- and resource-intensive activity that requires substantial staff time to be devoted to finding, auditing, and selecting local suppliers, and then mentoring or coaching their staff to improve their business processes.

Due to the frequent use of formal tendering in public procurement, public buyer–supplier relationships often do not have an explicit supplier development component built into them. Organizations such as SCMS and the USAID Global Health Supply Chain Program – Procurement and Supply Management Project (GHSC-PSM) have more flexibility in their sourcing and procurement practices to embed supplier development into their programs. To realize local sourcing objectives, specifically local firm growth, local economic impact, and program sustainability, multi-year programs/initiatives should adopt a strategic approach to local supplier development. They should also build in key performance metrics into health commodity procurement to measure the factors related to improving the quality of local labor force, local firm growth, and local economic impact.

Programs should adopt a strategic approach to local supplier development and use key performance metrics to measure growth and impact.

As a large share of health commodity procurement transitions to national government financing, it is important to embed local supplier development programs into the transition plans. It remains unclear if some of the benefits to local firms—such as easier access to local credit because of a fixed contract with a global organization or the reputational benefits associated with doing business with a global organization—would be as relevant when their main customer becomes the national government instead of a group like SCMS or GHSC-PSM. These are crucial areas of market and supplier development that require further examination.

## CONCLUSION

This study offers preliminary guidance on how bilateral and multilateral agencies could design effective local sourcing programs to create sustainable local markets for selected pharmaceutical products, laboratory, and transport services. Further investigation is required to better understand the procurement pathways that lead to local suppliers investing in new assets, institutionalizing new quality standards, recruiting new employees, or creating new service offerings.

## Supplementary Material

18-00083-Yadav-Supplement.pdf

## Notes

**Peer Reviewed**

**Funding:** PY and SA acknowledge research support from the William Davidson Institute at the University of Michigan for this study.
