# Supplementary material for: Local Sourcing and Supplier Development in Global Health: Analysis of the Supply Chain Management System's Local Procurement in 4 Countries
Source: Glob Health Sci Pract. 2018 Oct 3;6(3):574–83. doi: 10.9745/GHSP-D-18-00083 (PMC6172129; doi:10.9745/GHSP-D-18-00083)
Supplement: 18-00083-Yadav-Supplement.pdf [file 18-00083-Yadav-Supplement.pdf]

SUPPLEMENT. Supply Chain Management System Survey Tool

	**INTERVIEWER NAME:**		
	**DATE:**		
	**INTERVIEW START TIME:** hh:mm		
	**SECTION 1. CONTACT INFO**	**RESPONSE**	**NOTES/SKIP PATTERN**
1.1	Interviewee ID		
1.2	COMPANY NAME		
1.3	COMPANY ADDRESS		
1.4	May I begin the interview now?	1. □ YES<br>2. □ NO	Check one<br>*If "No", thank respondent and discontinue interview
**SECTION 2. ABOUT YOUR COMPANY**			
2.1	What is your position in this company?	1. □ Managing Director<br>2. □ Owner<br>96. □ Other (specify)______________________<br>98. □ Refused<br>99. □ Don't know	
2.2	What year was this company established?		

2.3	What type of company is this business registered as in this country?	1. □ Public<br>2. □ General Partnership<br>3. □ Limited Liability Partnership<br>4. □ Sole proprietorship<br>5. □ Government owned<br>6. □ Partially owned by government<br>96. □ Other (specify)__________________<br>98. □ Refused<br>99. Don't know	
2.4	Does this company have any branches in other parts of this country or abroad?	1. □ Yes<br>2. □ No<br>98. □ Refused<br>99. □ Don't know	Skip to question 2.7 if, "No", "Refused", "Don't know"
2.5	If yes, are the branches located in other cities within this country or abroad?	1. □ In other cities within this country<br>2. □ In other cities outside of this country<br>98. □ Refused<br>99. □ Don't know	
2.6	In what specific cities are the branches located?		
2.7	Have there been any ownership changes since the company was first established?	1. □ Yes<br>2. □ No<br>98. □ Refused<br>99. □ Don't know	Skip to question 2.9 if, "No", "Refused", "Don't know"
2.8	If yes, in what year?		
2.9	Have there been any changes in the management structure of this business since it was first established?	1. □ Yes<br>2. □ No<br>98. □ Refused<br>99. □ Don't know	Skip to question 2.11 if, "No", "Refused", "Don't know"

2.10	If yes, in what year?		
2.11	Does this company currently provide more, less or the same types of products and services compared to when it was first established?	1. □ More<br>2. □ Less<br>3. □ The same<br>98. □ Refused<br>99. □ Don't know	
2.12	When this company was first established, what services did it offer?	1. □ Procurement<br>2. □ Distribution<br>3. □ Warehousing<br>4. □ Printing<br>5. □ Network set-up<br>96. □ Other (specify1)____________<br>97. □ Other (specify2)____________<br>98. □ Refused<br>99. □ Don't know	more than one response allowed

2.13	When this company was first established, what products did it offer?	1. □ Lab Consumables<br>2. □ Lab Reagents<br>3. □ Lab Equipment<br>4. □ Pharmaceuticals (human)<br>5. □ Cold Chain for heat sensitive products<br>6. □ Electrical Equipment<br>7. □ IT equipment<br>8. □ IT supplies<br>9. □ Office Supplies<br>10. □ Handling Equipment of Warehouse<br>11. □ Pallets for Warehouse<br>12. □ Security equipment<br>13. □ Pharmaceuticals (animals)<br>96. □ Other (specify1)____________<br>97. □ Other (specify2)____________<br>98. □ Refused<br>99. □ Don't know	
2.14	What type of services does this company currently offer?	1. □ Procurement<br>2. □ Distribution<br>3. □ Warehousing<br>4. □ Printing<br>5. □ Network set-up<br>96. □ Other (specify1)____________<br>97. □ Other (specify2)____________<br>98. □ Refused<br>99. □ Don't know	more than one response allowed

2.15	What type of products does this company currently offer?	1. □ Lab Consumables<br>2. □ Lab Reagents<br>3. □ Lab Equipment<br>4. □ Pharmaceuticals<br>5. □ Cold Chain for heat sensitive products<br>6. □ Electrical Equipment<br>7. □ IT equipment<br>8. □ IT supplies<br>9. □ Office Supplies<br>10. □ Handling Equipment of Warehouse<br>11. □ Pallets for Warehouse<br>96. □ Other (specify1)_____________<br>97. □ Other (specify2)_____________<br>98. □ Refused<br>99. □ Don't know	
2.16	Approximate annual revenue?		
**SECTION 3: ABOUT SCMS CONTRACTS**			
3.1	Do you currently have an active contract with SCMS?	1. □ Yes<br>2. □ No<br>98. □ Refused<br>99. □ Don't know	
3.2	To date, how many SCMS contracts has your company been awarded?	1. □ one<br>2. □ two<br>3. □ three<br>4. □ four<br>5. □ five<br>96. □ other (specify)__________<br>98. □ Refused<br>99. Don't know	

3.3	A. In what year(s) did you have an active SCMS contract award?<br>B. What was the value of the award?<br>C. The contract equated to what % of company's annual revenue?	YEAR OF AWARD<br>1. □ 2007<br>2. □ 2008<br>3. □ 2009<br>4. □ 2010<br>5. □ 2011<br>6. □ 2012<br>7. □ 2013<br>96. □ Other (specify)___________________<br>98. □ Refused<br>99. □ Don't know	VALUE
3.4	PRODUCTS AND SERVICES UNDER CONTRACT AWARD 1	1. □ Lab Consumables<br>2. □ Lab Reagents<br>3. □ Lab Equipment<br>4. □ Pharmaceuticals<br>5. □ Cold Chain for heat sensitive products<br>6. □ Electrical Equipment<br>7. □ IT equipment<br>8. □ IT supplies<br>9. □ Office Supplies<br>10. □ Handling Equipment of Warehouse<br>11. □ Pallets for Warehouse<br>96. □ Other (specify1)____________<br>97. □ Other (specify2)____________<br>98. □ Refused<br>99. □ Don't know	
3.5	In order to fulfill the terms of your contract with SCMS, have you ever had to contract any other businesses (i.e, distributors/transporters, engineers, etc)?	1. □ Yes<br>2. □ No<br>98. □ Refused<br>99. □ Don't know	If "no", "refused", "don't know" skip to question 3.11

3.6	If yes, what type?	BUSINESS TYPE VALUE OF CONTRACT	
3.7	How many employees currently work here?	1. □ Less than 10<br>2. □ 10-24<br>3. □ 25-49<br>4. □ 50-74<br>5. □ 75-99<br>96. □ Other (specify)______________<br>98. □ Refused<br>99. □ Don't know	check one
3.8	Since the time you were first awarded an SCMS contract, has the number of employees working at this company increased, decreased, or stayed the same?	1. □ Increased<br>2. □ Decreased<br>3. □ Remained about the same<br>96. □ Other<br>98. □ Refused<br>99. □ Don't know	check one<br>Skip to question 3.12 if 'decreased', 'remained the same', 'refused', 'don't know'
3.9	By how many has the number increased/decreased?	1. □ Increased__________________<br>2. □ Decreased_________________<br>96. □ Other<br>98. □ Refused<br>99. □ Don't know	
3.10	To the best of your ability-please list all the positions in your company, education level, the number employed in those positions, required and the approximate salary range.	POSITION TYPE EDUCATION # EMPLOYED SALARY RANGE<br>1.<br>2.<br>3.<br>4.<br>5.<br>6.<br>7.<br>8.	

		9.<br>10.<br>11.<br>12.<br>13.<br>14.<br>15.	
3.11	Prior to being awarded the SCMS contract, did you offer any trainings to your employees?	1. □ Yes<br>2. □ No<br>98. □ Refused<br>99. □ Don't know	Skip to question 3.15 if "No", "Refused", "Don't know"
3.12	If yes, what type of trainings did you offer? How Frequently? # Trained per Session	TRAINING TYPE FREQUENCY #Trained<br>1. □ Management<br>2. □ Financial/Accounting<br>3. □ Safety<br>96. □ Other (specify1)____________________<br>97. □ Other (specify2)___________________<br>98. □ Refused<br>99. □ Don't know	
3.13	Did you start offering any new trainings after you were awarded the SCMS contract?	1. □ Yes<br>2. □ No<br>98. □ Refused<br>99. □ Don't know	Skip to SECTION 4 if "No", "Refused", "Don't know"
3.14	If yes, what type of additional trainings did you offer?How frequently? # Trained per session?	TRAINING TYPE FREQUENCY #Trained<br>1. □ Management<br>2. □ Financial/Accounting<br>3. □ Safety<br>96. □ Other (specify1)____________________<br>97. □ Other (specify2)___________________<br>98. □ Refused<br>99. □ Don't know	
3.15	Beyond the value of the contract, did SCMS offer any additional support to your company in the way of technical assistance, etc? (specify)	1. □ Yes<br>2. □ No<br>98. □ Refused<br>99. □ Don't know	
**SECTION 4. ABOUT THE MARKET LANDSCAPE**			

4.1	Have there been any changes in regulatory environment since your first SCMS contract award that directly affected your business?	1. □ Yes<br>2. □ No<br>98. □ Refused<br>99. □ Don't know	Skip to question 4.3 if "No", "Refused", "Don't know"
4.2	If yes, specify.		
4.3	The year that this company was awarded its first SCMS contract, approximately how many businesses existed that provided similar services as your company?	1. □ 1-4<br>2. □ 5-9<br>3. □ 10-14<br>4. □ 15-19<br>5. □ 20-25<br>96. □ other (specify)__________<br>98. □ Refused<br>99. Don't know	
4.4	Of those businesses providing similar services as your company, approximately how many did you consider to be direct competition?	1. □ 1-4<br>2. □ 5-9<br>3. □ 10-14<br>4. □ 15-19<br>5. □ 20-25<br>96. □ other (specify)__________<br>98. □ Refused<br>99. Don't know	
4.5	Approximately how many businesses ***currently*** exist in the market that provide similar services as your company?	1. □ 1-4<br>2. □ 5-9<br>3. □ 10-14<br>4. □ 15-19<br>5. □ 20-25<br>96. □ other (specify)__________<br>98. □ Refused<br>99. Don't know	

4.6	Approximately how many businesses ***currently*** exist in the market that you consider to be your direct competition?	1. □ 1-4<br>2. □ 5-9<br>3. □ 10-14<br>4. □ 15-19<br>5. □ 20-25<br>96. □ other (specify)_________<br>98. □ Refused<br>99. Don't know	
4.7	Since you were awarded the SCMS contract, would you say that your company has "grown", "stayed the same", or "decreased" in size and services offered?	1. □ The company has grown<br>2. □ The company has stayed about the same<br>3. □ The company has decreased<br>98. □ Refused<br>99. □ Don't know	Skip to SECTION 5 if "stayed the same", "decreased", refused", "don't know"
4.8	If your company has grown, would you say that your main competitors have grown as much?	1. □ Yes<br>2. □ No<br>3. □ Other companies have grown more<br>98. □ Refused<br>99. □ Don't know	
4.9	What percentage annual growth are you experiencing?		
**SECTION 5. ABOUT COMPANY ASSETS , ACCESS TO CREDIT, DEBT LEVELS**			
5.1	Did the number of tenders you won with other Government, bilateral, or multilateral agencies increase, decrease or stay the same after [SAY YEAR they were awarded first SCMS contract]?	1. □ Inceased<br>2. □ Decreased<br>3. □ Stayed the same<br>96. □ Other (specify))_________<br>98. □ Refused<br>99. □ Don't know	

5.2	Did the number of businesses you do regular business increase, decrease or stay the same after [SAY YEAR they were awarded first SCMS contract]?	1. □ Inceased<br>2. □ Decreased<br>3. □ Stayed the same<br>96. □ Other (specify))__________<br>98. □ Refused<br>99. □ Don't know	
5.3	What are the major new accounts you have won since [SAY YEAR they were awarded first SCMS contract]? (specify year)	NAME<br>YEAR<br>1.<br>2.<br>3.<br>4.<br>5.<br>6.<br>7.	
5.4	Approximately how many businesses do you do regular business with in a given year?	1. □ Less than 10<br>2. □ 10-20<br>3. □ 21-30<br>4. □ 31-40<br>5. □ 41-50<br>6. □ 100<br>7. □ 200<br>96. □ Other (specify)__________<br>98. □ Refused<br>99. □ Don't know	

5.5	Where does SCMS rank among your biggest clients in terms of contract value?	1. □ SCMS is our biggest client<br>2. □ Top 5<br>3. □ Top 10<br>96. □ Other (specify)_________<br>98. □ Refused<br>99. □ Don't know	
5.6	Since [SAY YEAR they were awarded first SCMS contract], do you think your company's reputation has increased, decreased or stayed the same?	1. □ Inceased<br>2. □ Decreased<br>3. □ Stayed the same<br>96. □ Other (specify)_________<br>98. □ Refused<br>99. □ Don't know	
5.7	Since [SAY YEAR they were awarded first SCMS contract], do you find that you are in a better, worse or same position to negotiate prices with your suppliers?	1. □ Better<br>2. □ Worse<br>3. □ The same<br>96. Other (specify)________________<br>98. □ Refused<br>99. □ Don't know	
5.8	Has the number of assets your company owns (e.g, vehicles, forklifts, office space, warehouses, machinery, etc.) increased, decreased or stayed the same since [SAY YEAR they were awarded first SCMS contract]?	1. □ Inceased<br>2. □ Decreased<br>3. □ Stayed the same<br>96. □ Other (specify)_________<br>98. □ Refused<br>99. □ Don't know	
5.9	Estimated value of new assets obtained since [SAY YEAR they were awarded first SCMS contract]?		

5.10	Has your company's ability to access credit, loans, bank facilities, increased, decreased or stayed the same since [SAY YEAR they were awarded first SCMS contract]?	1. □ Inceased<br>2. □ Decreased<br>3. □ Stayed the same<br>96. □ Other (specify)_________<br>98. □ Refused<br>99. □ Don't know	
5.11	While working to fulfill the SCMS contract, were monies owed to you by SCMS ever delayed (i.e., paid outside of agreed payment period)?	1. □ Yes<br>2. □ No<br>3. □ On some occasions<br>98. □ Refused<br>99. □ Don't know	
5.12	While working to fulfill the SCMS contract, were you able to pay your suppliers on time?	1. □ Yes<br>2. □ No<br>98. □ Refused<br>99. □ Don't know	Skip to SECTION 6 if "Yes", "Refused", "Don't know"
5.13	If no, why not?		
**SECTION 6: ABOUT YOUR COMPANY'S EFFICIENCY/PRODUCTIVITY**			
6.1	Have you made investments in business systems, software to improve operations?	1. □ Yes<br>2. □ No<br>98. □ Refused<br>99. □ Don't know	
6.2	If yes, of what nature?		
6.3	Have you established new standard operating procedures?	1. □ Yes<br>2. □ No<br>98. □ Refused<br>99. □ Don't know	
6.4	If yes, for what? (when?)		
6.5	Any final comments		
	**THANK RESPONDENT AND END INTERVIEW**		
